# An Integrative Review of Interprofessional Teamwork and Required Competence in Specialized Palliative Care

**DOI:** 10.1177/00302228221085468

**Published:** 2022-04-19

**Authors:** Pauliina Kesonen, Leena Salminen, Johanna Kero, Johanna Aappola, Elina Haavisto

**Affiliations:** 1 Department of Nursing Science, 8058 University of Turku, Turku, Finland; 2 Turku University Hospital, Turku, Finland; 3 Satakunta Hospital District, Pori, Finland

**Keywords:** palliative care, competence, review, interprofessional care, patient care team

## Abstract

To deliver quality care, social and healthcare professionals should be competent both in their own professional work and interprofessionally. The aim of this integrative review was to describe interprofessional teamwork and the required competencies for teamwork in specialized palliative care. Totally 14 studies published between 2003 and 2020 were included in the review. Interprofessional teamwork was described from the patients and professionals’ perspective. The required interprofessional competencies were described as teamwork knowledge, skills, attitudes, and values. Interprofessional teamwork is one of the essential factors in providing holistic and ethically sustainable care to palliative patients. The way how professionals confront death and dying effects the whole team; this suggests that support practices are important in palliative care settings. Ascertaining the interprofessional competence in palliative care will produce better collaborative practices and increase the care outcomes. The findings can be used as a framework when developing interventions to promote clinical and educational practices.

## Introduction

The need for palliative care is increasing globally because of the aging population and health issues that have become progressively complex. It has been estimated that every year over 56,8 million people in the world need palliative care but only 12% are actually receiving it (World Palliative Care Alliance & World Health Organization [WPCA & WHO], 2020) Palliative care delivery varies in different countries, and it can be implemented in different kinds of clinical settings*.* In specialized palliative care, the expertise of multiple different professionals is used to treat patients more holistic way; when the care needs are complex, intense, and required more often (Buss et al., 2017; Gamondi et al., 2013).

In palliative care settings, holistic care is highlighted and attitudes toward medical care as being the center of palliative care are changing (Buss et al., 2017). Collaborative working has become core value when providing high-quality palliative care (European Association for Palliative care [EAPC], 2020; WHO, 2016) because it gives a better perspective on patients’ diverse health-related issues (Hui et al., 2018). There are various concepts regarding professional teamwork in social and healthcare settings (Reeves et al., 2017; Thylefors et al., 2005). Despite previous studies and definitions, in the literature, the use of these terms is either not systematic, or no definition of the terms is provided (D’Amour et al., 2005; Reeves et al., 2017; Thylefors et al., 2005). When referring to different professionals working together, the concepts of inter-, multi-, and trans-professional are commonly used (Thylefors et al., 2005). The term “discipline,” however, refers to a more theoretical notion (D’Amour et al., 2005; Mahler et al., 2014). The term “profession” involves scientific knowledge of a specific discipline and describes an occupation in a practical manner. In this article, the term “interprofessional” was chosen because it refers to collaborative working where different professionals work directly together with the aim of ensuring the type of care in which patients receive benefits from each profession. (Mahler et al., 2014.) In this study, interprofessional teamwork has been defined as social and healthcare professionals with different educational backgrounds working together to deliver high-quality palliative care at a specialized level.

To accomplish effective palliative care in interprofessional teams, professionals must master interprofessional competencies as well as discipline specific competencies (Witt Sherman et al., 2017). Interprofessional competencies are those which all professionals need when working with other professionals, patients, families, and organizations (IPEC, 2016). Different professionals have their own specific culture, language, knowledge, and skills and when professionals work together, actions are needed to sustain their collaborative working (Wilhelmsson et al., 2012). However, receiving little or no training is not unusual (WHO, 2016). In this review, competence is defined according to Meretoja (2003) as a “functional adequacy and the capacity to integrate knowledge, skills, attitudes and values in specific contextual situations.” Using this definition, a coherent description of the demands of competence was achieved that was not dependent on the individual professional nor the educational background of the team members. Different competence frameworks have been developed as regards palliative care (Connolly et al., 2016; Gamondi et al., 2013) and interprofessional teamwork (Canadian Interprofessional Health Collaborative [CIHC], 2010; D’Amour et al., 2005; IPEC, 2016; WHO, 2010; Wilhelmsson et al., 2012; Wood et al., 2009). Additionally, professional specific competencies have been developed for multidisciplinary hospice and palliative care professionals in order to identify the roles of each specialist involved in a care team (Kang et al., 2013).

When developing care delivery, required competencies need to be defined. To our best knowledge previous literature does not define the required interprofessional competence for palliative care professionals that would facilitate the promotion of interprofessional teamwork and increase the quality of care for patients who are in a need for more specialized palliative care. Therefore, the aim of this literature review was to describe interprofessional teamwork in specialized palliative care settings and to describe the required competencies for teamwork. The ultimate goal is to promote high-quality palliative care by producing knowledge to develop collaborative practices. The research questions were as follows: (1) What is interprofessional teamwork in specialized palliative care? and (2) What are the required competencies in interprofessional teamwork in specialized palliative care?

## Methods

### Design

An integrative literature review was chosen to describe interprofessional teamwork and required competence in specialized palliative care settings, because it allowed the use of studies conducted with different methodologies in the area which is little studied. Attention was paid when combining different methodologies because of the possible lack of rigor, potential biases, and inaccuracies; thus, systematic, and precise data analysis methods were used. This integrative review was conducted in five stages (Whittemore & Knafl, 2005): 1. The problem was identified to set clear purposes for the review; 2. The literature was searched to find relevant studies from suitable databases; 3. The data was evaluated to indicate the quality of the existing studies; 4. The data analysis was converted into the groupings: organize, code, categorize; 5*.* A synthesis of the results was made followed by a conclusion.

### Literature search

A systematic literature search was conducted in February 2021 using the following databases: Cochrane, PubMed, CINAHL, Scopus, and Web of Science. The time period chosen were the years between 2003 and 2021, as according to the Council of Europe (2003) all members needed to have a coherent and consistent framework for palliative care delivery. The recommendations contained elements such as facilitating active participation by palliative patients in their own care and meeting the patient with dignity and respect.

The inclusion criteria were 1) peer-reviewed original studies with an available abstract, 2) studies concerned with the interprofessional teamwork of social and healthcare professionals working with specialized palliative care for adult patients, and 3) studies in which nursing professionals were named as part of the team. The exclusion criteria were 1) studies of students and non-qualified social or healthcare professionals including volunteers, 2) studies conducted in primary care settings, like nursing homes and assisted living, 3) studies measuring the effectiveness of interprofessional educational interventions. The PICO model was utilized to specify the search terms and construct the search strategy (Schardt et al., 2007). The search terms used with the Boolean operators are presented in the Table 1 with an example of the PubMed search. The search phrases were formed according to the requirements of each database. The search was conducted in collaboration with two information specialists.

**Table 1.** *PICO strategy complemented with search terms*.

Pico element	Keywords	Search terms (example: PubMed)
P (Patient or Population)	Palliative care	(Palliative care* OR palliative nurs* OR “Hospice and Palliative Care Nursing”[Mesh] OR “Terminally Ill”[Mesh] OR “Terminal Care”[Mesh] OR “end of life” OR life-limiting* OR dying* OR “Hospice Care”[Mesh] OR “Hospices”[Mesh] OR non-curative*)
		**AND**
I (Intervention)	interprofessional teamwork, collaboration, (requirement: nursing professionals are part of the care team)	(teamwork* OR “Patient Care Team”[Mesh] OR collaborat* OR co-operat* OR “palliative care team” OR “palliative care teams” OR “Interprofessional Relations”[Mesh] OR “Interdisciplinary Communication”[Mesh] OR “Interdisciplinary Research”[Mesh] OR interprofession* OR interdisciplin* OR multiprofession* OR multidisciplin* OR transdisciplin*)
		**AND**
		(“Nurses”[Mesh] OR “Nurse Practitioners”[Mesh] OR “Nurse Specialists”[Mesh] OR “Licensed Practical Nurses”[Mesh] OR “Nurses, Public Health”[Mesh] OR nurs* OR registered nurse* OR staff nurse* OR enrolled nurse*)
		**AND**
C (Comparison or control)	—	—
O (Outcome)	Competence	(competenc* OR “Professional Competence”[Mesh] OR “Clinical Competence”[Mesh] OR knowledge* OR skill* OR professional skill* OR clinical skill* OR abilit* OR readiness* OR prepared* OR require*)

**Figure 1.**
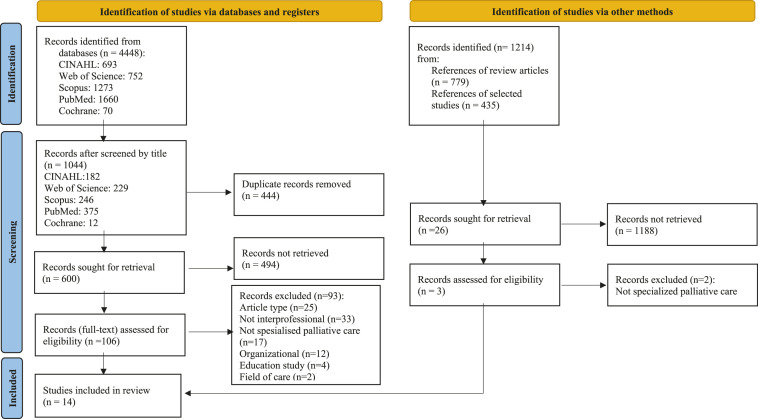
*Progression of the literature search*.

As a result, 4448 studies were identified and screened by their title (Figure 1). One researcher performed a headline scan independently. Additionally, 1214 records were identified through a manual search of the references in the review articles and selected studies. After removing duplicates, a total of 626 studies were screened by their abstract. RefWorks reference management software was used to manage the references. Abstract screening was conducted together with two reviewers (PK & JA). Two reviewers then assessed the full texts of the chosen articles (n=109), first independently and then together. A third reviewer (EH) was used to solve ambiguities with unclear articles. The PRISMA 2020 flow diagram for systematic reviews was followed for transparent reporting (Page et al., 2021) and the most common reasons for exclusions are presented in Figure 1.

*From:* Page, M. J. et al., (2021). The PRISMA 2020 statement: an updated guideline for reporting systematic reviews. *BMJ (Clinical research ed.)*, *372*, n71. https://doi. org/10.1136/bmj.n71.

### Quality Appraisal

To assess the methodological quality of the chosen studies Joanna Briggs Institutes Critical Appraisal Tools were used. The included lists of questions with a four-item scoring: “yes, no, unclear, not applicable”; scores were only given to “yes” answer to assess the trustworthiness, relevance, and results of the studies. (Joanna Briggs Institute [JBI], 2017.) The quality of the studies was assessed independently by two reviewers (PK & JK). The assessments were compared, and differences were resolved by discussion. The aim of the data evaluation was to describe the quality of the chosen studies, rather than exclude any studies because of poor quality; therefore, no studies were excluded (Table 2).

**Table 2.** *Description of the studies reviewed (n = 14)*.

Author/year/country	Aim	Study design/ measurement/instrument	Setting	Participants	Main results	JBI scores
1. Brennan et al. (2016), United States	To improve efficiency of interprofessional team meetings and improve communication among different specialties	A quasi-experimental pretest–posttest study before and after PDSA cycles, Stakeholder survey	One palliative care team	Chaplains, medicine, nursing, nutrition, pharmacy, social work, speech pathology and research (n=14)	Prioritizing meeting topics and scheduling them is an ongoing challenge. Clear structure in the meeting is essential, but when working collaboratively, flexibility is needed. Intervention increased team cohesion and participation in all professions.	6/9 ^1)^
2. Demiris et al. (2008),United States	Understand interactions of hospice providers during IDT meetings and explore how meetings contribute the function of team	Qualitative exploratory study, videotaped team meetings/patient care discussions (n = 81)	Four hospice teams	Physicians, nurses, social workers, bereavement counselors, chaplains, volunteer co-ordinators and home health aides (n = 5–12 members in a team)	Nurses had the most active role in team functioning. Enhancement was needed to improve team member support and effectiveness of communication. Professionals receive little or no training on communication in interprofessional teams.	7/10 ^2)^
3. Goebel et al. (2016), United States	Describe palliative care team structures, teamwork processes and perceptions of quality of care and to examine how team structures influence on perceptions of quality of life	Cross-sectional, online survey including demographic and organizational questions and TeamSTEPPS questionnaire	Palliative care professionals in five countries	Nurses (n = 373) and other professionals, e.g., physicians, chaplains, social worker (n = 87)	Communication, collaboration, and diversity of the team were important factors in quality of care. Also, opportunity to collaborate with several disciplines was significantly associated with perceptions of excellent quality of care. Work environment was felt to be hierarchical. Possibility to join interprofessional education is related to academic settings and it was reported as a challenge. It is important to have possibility to have more education while working.	4/8 ^3)^
4. Jünger et al. (2007), Germany	Identify relevant factors considering teamwork in palliative care and identify the criteria which enhance co-operation in the team	Qualitative exploratory study, Semi-structured open interviews	One palliative care team	Nurses, psychologists, physicians, and secretaries (n=19)	Communication, team philosophy, high team commitment, autonomy and good interpersonal relationships in a team were identified as components of good teamwork. Also dealing with death was seen essential when working in palliative care. Unclear goals and role delegation were an inefficient way to work in teams. The team is seen as important when dealing with death and dying.	7/10 ^2)^
5. Klarare et al. (2013), Sweden	Explore team members interaction in specialized palliative home care teams	Qualitative exploratory study, semi-structured interviews	Five specialized palliative homecare teams	Physicians, nurses, paramedical staff, and social workers (n=15)	Knowledge and trust in other professions’ competences strongly effects team performance and atmosphere. Communication is the key element in a team. Leadership and organizational issues have great impact on palliative care delivery.	6/10 ^2)^
6. Leboul et al. (2017), France	Provide comprehensive description of healthcare providers options about sedation practices	Qualitative descriptive study, multi-professional focus group interviews and written narratives	Three palliative care units in palliative care and university hospitals	Physicians, nurses, assistant nurses, psychologists, physiotherapists, psychomotor therapists, and volunteers (n = 35)	Professional competence and stance played a major role when using sedation in palliative care. Healthcare teams had a key role providing support and minimizing the suffering caused by the work itself. Palliative sedation is felt to be a care situation where discussion and team support are highly valued. The possibility to discuss matters was related to the quality of care.	7/10 ^2)^
7. Mélin et al. (2020), France	Identify the used practices to support relatives and understand their expectations considering support during their agonal phase in palliative care units	Qualitative exploratory study, focus group interviews	One palliative care unit	Nurses, care assistants, doctors, psychologists, volunteers, psychometrician, secretary, socio-educational assistant, socio-esthetician, health executive, physiotherapists, hospital service agent (n = 25) and relatives (n = 7)	In addition to quality care being provided for the patient, giving support to the relatives is important. Four support practice areas were identified: Providing care and ensuring comfort; communicating, informing, and explaining; interacting and mobilizing interdisciplinary skills in a team.	3/10 ^2)^
8. O'Connor & Fisher (2011), Australia	Explore team members experiences and perceptions considering team dynamics	Qualitative exploratory study, semi-structured interviews	Three palliative care sites	Palliative care nurses, palliative care medical specialists, a psychiatrist, social worker, counselor, and occupational therapists (n=7)	Clear role boundaries and the maintenance of those boundaries are both needed in palliative care to facilitate teamwork among different professionals. With team training programs and policies but also adequate resources, it is possible to enhance effective teamwork.	8/10 ^2)^
9. Seow & Bainbridge (2018), Canada	Examine how home-based specialized palliative care teams created their team and identify critical steps during their development	Descriptive qualitative design, semi-structured group interviews and other documentation	15 specialized palliative care teams	Nurses, physicians, personal support workers, spiritual counselors, and administrators (n = 122, ranging from 4 to 10 per team)	There are four different stages (Inception, Start-up, Growth and Mature) in the development of palliative care teams from inception to mature teams. When these phases are recognized, it may assist planners and workers to understand the process, the amount of time needed and the determination necessary. Time is required when building a team with different professionals.	7/10 ^2)^
10. Sundus et al. (2018), Malaysia	Investigate views of healthcare professionals regarding integration of pharmacist into palliative care team	Qualitative exploratory study, semi-structured interview with open-ended questions	Cancer palliative care team at oncology and palliative care ward in public hospital	Physicians, oncologists, nurses, pharmacists, patients, and caregivers (n = 29)	Attendance of pharmacists improves patient-centered care. In addition to which the additional knowledge about drugs and drug-related problems was highly welcomed by other professionals. Compared to older studies, professionals had a positive response to adding pharmacists into their team and they knew what kind of effort to expect from pharmacists.	7/10 ^2)^
11. Washington, et al. (2017a), United States	Study information sharing in hospice interdisciplinary group meetings to develop informatic tools to support collaboration	Qualitative descriptive study, individual interviews, and video recordings of IDG meetings	Hospice agencies	Nurses, chaplains, physicians, social workers, bereavement counselors and volunteer co-ordinators (n = 24) and patients’ care plan discussions (n = 57)	To deliver holistic care and meet patient and family’s goals, the sharing of physical, psychosocial, and spiritual information is necessary. Informatic tools in meetings are essential to summarize information and help professionals to convert the data into usable knowledge.	7/10 ^2)^
12. Washington et al. (2017b), United States	Understand hospice providers perspectives and experiences about interprofessional meetings; facilitators and barriers to effective team functioning	Exploratory case study, individual interviews	11 different hospice agencies in 7 states	Nurses, chaplains, social work, medical doctors, bereavement counselors and volunteer co-ordinators (n = 24)	Commitment and understanding different perspectives were identified as facilitators. Professionals felt frustrated under demanding caseloads and inefficiencies. In addition, time pressure and missing attendees were identified as barriers. Standardizing interprofessional meetings could enhance team functioning and efficiency.	7/10 ^2)^
13. Washington et al. (2016), United States	Describe shared decision-making in interdisciplinary team meetings	Exploratory descriptive study with video recordings of team meetings, professionals and family care givers individual interviews and research field notes.	5 hospice teams	Video recordings (n = 100), professionals e.g., nurses, chaplains, physicians, and social workers (n = 78) and family caregivers (n = 73) interviews	Time constraints, staff absence, communication skills and unclear role expectations were barriers in interdisciplinary meetings. Shared decision-making did not commonly occur in meetings that included family caregivers. Using videoconferencing creates a platform for shared decision-making in hospice interdisciplinary team meetings. Work needs to be done to allow family caregivers to be a part of the team and have an equal status as decision-makers.	7/10 ^2)^
14. Wittenberg-Lyles et al. (2010), United States	Explore team members perceptions of collaboration and collaborative communication practices in interdisciplinary team meetings	Mixed-method study with videotaped team meetings and MIIC instrument	Two hospice team	Nurses (n=17), social workers (n=3), chaplains (n = 3), medical directors (n = 2), hospice caregivers (n = 13) and miscellaneous group members, for example, volunteer co-ordinators and medical students (n = 18)	Collaborative practice and shared learning were promoted when professionals had the possibility to share and reflect on the cases. Team members reported to have perceptions of the flexibility and interrelation of roles. Caregivers participation had a positive influence on collaborative communication in palliative care team meetings and caregivers presence had an impact on the reflective processes in meetings.	6/10 ^2)^ 6/8 ^4)^

Used Joanna Briggs Institute Critical Appraisal tools: Checklists for Quasi-Experimental Studies ^1)^, Qualitative Research ^2)^, Analytical Cross-Sectional Studies^3)^, Case Reports^4)^

A relevant tool was selected according to the methodology utilized in the chosen study. Checklists for Quasi-Experimental Studies, Qualitative Research, Analytical Cross-Sectional Studies and Case Reports were used. The checklist for Quasi-Experimental Studies assesses the description of comparisons, existence of comparison group, used measurements and statistical analyses. The checklist for Qualitative Research assesses the congruity among philosophical perspective, research methodology and analysis methods, but also researchers influence on the study and the presentation of participant voices. The checklist for Analytical Cross-Sectional Studies assesses the description of study subjects and setting, issues considering confounding factors, used measures and statistical analyses. The checklist for Case Reports assesses the description of participant characteristics, tests/assessment methods, results, and both intervention and post-intervention condition. (JBI, 2017.)

### Data Analysis

During the analysis, data from the primary articles was organized, categorized, and summarized into an integrated conclusion according to the aim of the review and research questions (Whittemore & Knafl, 2005). Original expressions were identified from primary sources according to their relevance rather than their frequency. Research question one (1) studies were analyzed using inductive content analysis. (Elo & Kyngäs, 2008.) After reading the studies and becoming familiarized with the content, the original expressions were reduced and identified as subcategories. Subcategories with similarities were grouped into main categories describing interprofessional teamwork in specialized palliative care (Table 3).

**Table 3.** *Inductive category forming (research question 1)*.

Main category	Category	References
Patient perspective on interprofessional palliative care	Ethically approved palliative care	Klarare et al. (2013); Leboul et al. (2017); Sundus et al. (2018); Mélin et al. (2020)
	Individual palliative care	Brennan et al. (2016); Leboul et al. (2017); Washington et al. (2017a); Washington et al. (2017b); Sundus et al. (2018)
	Holistic palliative care	O'Connor and Fisher (2011); Klarare et al. (2013); Washington et al. (2017a); Washington et al. (2017b); Sundus et al. (2018)
	Communication with palliative care patients and their families	Washington et al. (2017b); Sundus et al. (2018); Mélin et al. (2020)
Professionals’ perspective on interprofessional palliative care	Understanding the nature of palliative care	Jünger et al. (2007); Klarare et al. (2013); Leboul et al. (2017)
	Team cohesion	Jünger et al. (2007); Demiris et al. (2008); Klarare et al. (2013); Brennan et al. (2016); Washington et al. (2016); Leboul et al. (2017); Washington et al. (2017b)Washington, Guo, et al. (2017); Seow & Bainbridge (2018); Mélin et al. (2020)
	Shared goals	Jünger et al. (2007); Demiris et al. (2008); Brennan et al. (2016); Seow & Bainbridge (2018)
	Professional contribution	Jünger et al. (2007); Demiris et al. (2008); Wittenberg-Lyles et al. (2010); O'Connor and Fisher (2011); Klarare et al. (2013); Brennan et al. (2016); Goebel et al. (2016); Leboul et al. (2017); Washington et al. (2017a); Washington et al. (2017b); Seow & Bainbridge (2018); Mélin et al. (2020)
	Work management	Klarare et al. (2013); Brennan et al. (2016); Washington et al. (2016); Washington et al. (2017b)

Research question two (2) was analyzed using deductive content analysis based on Meretoja´s definition of the four competence areas: knowledge, skills, attitudes, and values (Meretoja, 2003). After reviewing the data, the content was divided into the identified categories (Table 4) (Elo & Kyngäs, 2008).The analysis was conducted by one researcher, after which the composition and understandability were discussed and verified by the research group.

**Table 4.** Deductive category forming (research question 2).

Competence area	Subcategory	References
Knowledge	Roles and responsibilities in a team	Brennan et al. (2016); Seow & Bainbridge (2018); Sundus et al. (2018)
	Other professionals’ expertise	O'Connor and Fisher (2011); Klarare et al. (2013); Brennan et al. (2016); Sundus et al. (2018); Mélin et al. (2020)
	Own expertise	Demiris et al. (2008); O'Connor and Fisher (2011); Klarare et al. (2013); Sundus et al. (2018)
Skills	Co-operation	Wittenberg-Lyles et al. (2010); Brennan et al. (2016); Goebel et al. (2016); Sundus et al. (2018)
	Communication	Jünger et al. (2007); Demiris et al. (2008); Klarare et al. (2013); Leboul et al. (2017); Washington et al. (2017a); Washington et al. (2017b); Seow & Bainbridge (2018); Sundus et al. (2018)
	Flexibility	Jünger et al. (2007); Brennan et al. (2016); Leboul et al. (2017); Washington et al. (2017b); Seow & Bainbridge (2018); Mélin et al. (2020)
	Effectivity	Demiris et al. (2008); Klarare et al. (2013); Brennan et al. (2016); Washington et al. (2017b)
	Ability to evolve	Jünger et al. (2007); Demiris et al. (2008); Seow & Bainbridge (2018)
Attitudes	Commitment	Jünger et al. (2007); Demiris et al. (2008); O'Connor and Fisher (2011); Brennan et al. (2016); Washington et al. (2017b); Seow & Bainbridge (2018)
	Trust	Jünger et al. (2007); Demiris et al. (2008); O'Connor and Fisher (2011); Sundus et al. (2018)
	Being supportive	Jünger et al. (2007); Demiris et al. (2008); Klarare et al. (2013); Leboul et al. (2017); Seow & Bainbridge (2018)
Values	Respect	Jünger et al. (2007); Washington et al. (2016); Washington et al. (2017b); Sundus et al. (2018); Mélin et al. (2020)
	Equality	Washington et al. (2016); Washington et al. (2017b)

## Results

### Description of the Studies

Fourteen of the articles met the inclusion criteria of which 11 were conducted with a qualitative approach; one of the studies was a mixed method. All articles were published in English (Table 2). The studies were conducted from 2007 to 2020 and most were carried out in Northern America (n=8), seven of which were in the United States. The others were in Europe (n=4), Australia (n=1) and Asia (n=1) (Table 2). There was variation to some extent regarding how the characteristics of participants were presented in the included studies. Six of the studies did not provide any information about the participating professionals, except the name of their profession (Brennan et al., 2016; Demiris et al., 2008; Sundus et al., 2018; Washington et al., 2016, 2017a, 2017b). One study provided the characteristics of the participants in a vague format that could not be reported in this review (Leboul et al., 2017). Participant characteristics are presented in Table 5.

**Table 5.** Participant characteristics in included studies.

Study	Age (years)	Gender	Education	Working experience in in clinical practice (years)	Working experience in pc (years)
Goebel et al. (2016)	26–76 Mean 51.9 SD 9.7	Male (n = 40) Female (n = 357)	Less than Bachelor 57 (14.2) Bachelor 86 (SD 21.4) Master 196 (48.9) Doctoral 62 (15.5)	0.5–49 Mean 22.6 SD 12.3	0–38 Mean 9.3 SD 7.3(months)
Jünger et al. (2007)	35–49 Mean 40.4 SD 4.2	Male (n = 6) Female (n = 13)	—	—	Mean 10.53 SD 4.07
Klarare et al. (2013)	30–40 (n = 1) 41–50 (n = 5) 51–60 (n = 6) 61–70 (n = 3)	Male (n = 4) Female (n = 11)	—	—	5–8 (n = 5) 9–14 (n = 5) 15–20 (n = 2) >30 years 20?> (n = 3)
Mélin et al. (2020)	—	—	—	—	3 months–20 years Mean 8 (physicians) and 3 (all other staff)
O'Connor & Fisher (2011)	35–56 Mean 46.4	Male (n = 3) Female (n = 4)	—	—	7–20 Mean 12
Seow & Bainbridge (2018)	—	Male (n = 20) Female (n = 97)	—	—	0–5 (n = 53) 6–10 (n = 23) 11< (n = 28) Unknown (n = 18)
Wittenberg-Lyles et al. (2010)	Mean 61.5	Male (n = 12) Female (n = 44)	—	—	—

Concepts regarding different professionals working together were only clearly defined in three studies (Jünger et al., 2007; Klarare et al., 2013; Wittenberg-Lyles et al., 2010). It seems there is no coherent framework available that defines the requirements for participating in interprofessional teams aiming to provide ideal holistic care for patients in specialized palliative care. In every study, the nursing and medical staff participated in the interprofessional teamwork, but the participation of other professionals varied (Table 6). Patients were included in one study and family caregivers in two studies, with their role varying from being a participant to an active member of the care team.

**Table 6.** Professionals’ participation in interprofessional teamwork in the selected studies.

Profession	Relevance (n)
Nursing	14
Medicine	14
Social work	9
Spiritual care	7
Bereavement counselors	3
Psychologists	3
Pharmacists	2
Secretaries	2
Physiotherapists	2
Other counselors	2
Psychomotor therapists, occupational therapists, nutritional experts, speech pathologists, hospice care givers, home health aides, paramedical staff, personal support workers, administrators, medical students, psychometrician, socio-educational assistant, socio-esthetician, health executive, hospital service agent, and research persons	1

Checklists for Qualitative Research (n=11), Quasi-Experimental Studies (n=1) and Analytical Cross-Sectional Studies (n=1) were used (Table 2). One mixed-method study was evaluated using two different tools, Case Reports and Qualitative Research. In general, the scores received were good. Congruity between the research methodology, research questions or objectives, data collection methods, data analysis and representation were well reported. In addition, the participants’ voice was presented well and the studies followed the current ethical criteria. The most commonly missing element as regards the studies was the reporting of qualitative methods and addressing the researchers influence on the research. Additionally, the philosophical perspective of congruity compared to the research methodology was unclearly described in most of the studies. In the quasi-experimental study, the control group was missing thus affecting the causal inferences of the study.

### Interprofessional Teamwork in Specialized Palliative Care

**Figure 2.**
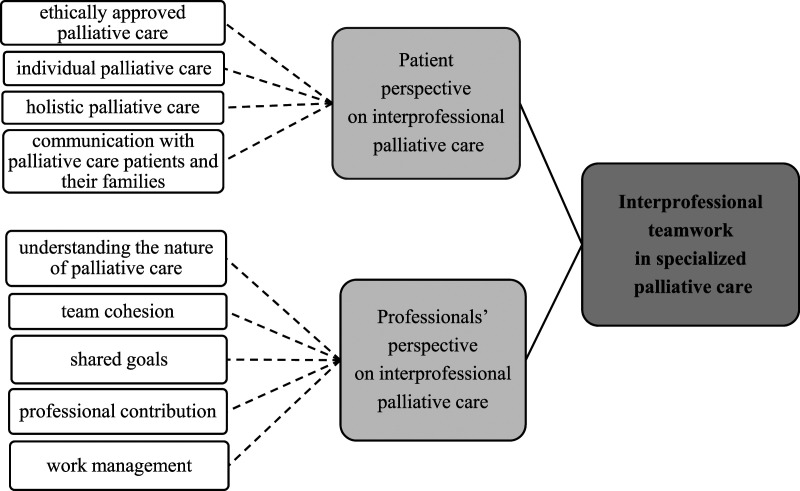
*Interprofessional teamwork in specialized palliative care*.

Interprofessional teamwork in specialized palliative care can be described from the patients and the professionals’ perspective (Figure 2). The patient´s perspective on interprofessional palliative care was described as *ethically approved palliative care, individual palliative care, holistic palliative care,* and *communication with palliative care patients and their families*. *Ethically approved palliative care* means ensuring patients’ dignity and autonomy and respecting the patient when in care (Klarare et al., 2013). In addition, providing relief (Leboul et al., 2017; Mélin et al., 2020) and ensuring safety as a part of care is essential (Sundus et al., 2018). *Individuality in palliative care* means delivering patient-centered care (Sundus et al., 2018) by noticing the diverse (Washington et al., 2017a) and individual care needs of patients (Brennan et al., 2016; Leboul et al., 2017; Washington et al., 2017b) when a patient has a life-limiting disease. *Holistic palliative care* is seen as the core of palliative care; therefore, it is important to treat the patients holistically (Klarare et al., 2013; Washington et al., 2017b) and understand different contexts of their life which affect their care (O'Connor & Fisher, 2011) and to meet both the patients’ and their family’s goals in the care provided (Washington et al., 2017b). Interprofessional meetings are one way to maintenance holistic care plans to patients and their family members (Mélin et al., 2020). To manage patients’ symptoms (Sundus et al., 2018; Washington et al., 2017b) information about emotional well-being is as important as information concerning the illness itself (Washington et al., 2017b). *Communication with palliative care patients and their families* is seen as the responsibility and capability of every professional in an interprofessional team. Communication consists of giving individual counseling, providing relief (Sundus et al., 2018) and generally discussing matters with both the patient and family (Washington et al., 2017a), formally and informally (Mélin et al., 2020). Devoting time by being present and listening are important in palliative care settings (Mélin et al., 2020).

The professionals’ perspective on interprofessional palliative care was described as *understanding the nature of palliative care, team cohesion, shared goals, professional contribution,* and *work management.* In interprofessional teamwork in specialized palliative care it is essential to *understand the nature of palliative care*. The ability of each professional to confront and deal with death and dying influences the whole interprofessional team working in palliative care. It is also important to deal with one’s own feelings when facing death (Jünger et al., 2007; Klarare et al., 2013), because palliative care is complex branch of care (Klarare et al., 2013). It is a natural part of palliative care that professionals are also present when patients are dying. Sometimes professionals might be afraid of causing or being responsible for the patients’ death. (Klarare et al., 2013; Leboul et al., 2017.)

*Team cohesion* is about sustaining the feeling of being a team, so that members are integrated (Seow & Bainbridge, 2018) with a joint philosophy (Jünger et al., 2007) and values (Mélin et al., 2020). Every member of the team should adopt the team culture (Mélin et al., 2020) by supporting (Washington et al., 2017a), serving and helping other professionals (Seow & Bainbridge, 2018). Team support is important (Demiris et al., 2008) and it reduces the burden the team is faces in their work (Leboul et al., 2017). Determining shared rules and general outlines is an important aspect of teamworking (Leboul et al., 2017; Washington et al., 2017a); therefore, independent operators are seen as being disadvantaged as regards collaboration (Brennan et al., 2016; Washington et al., 2016). A common language is one of the important elements to co-operate successfully (Klarare et al., 2013). A positive atmosphere (Jünger et al., 2007) and a sense of team spirit needs to be built and consistently maintained (Brennan et al., 2016; Demiris et al., 2008; Klarare et al., 2013). Every member should feel that they are part of the team and should be allowed to participate (Brennan et al., 2016). Standardized interprofessional processes can help to increase job satisfaction and reduce negativity in teams (Washington et al., 2017a) as can designating a team leader to keep the team working (Klarare et al., 2013; Washington et al., 2016). Collaboration in- and outside of formal working hours is important (Mélin et al., 2020; Washington et al., 2017a); for example, common support groups might be a functional solution (Klarare et al., 2013). In interprofessional teams, it is essential that professionals have a common understanding of the *shared goals* (Brennan et al., 2016; Jünger et al., 2007; Seow & Bainbridge, 2018). Moreover, well-defined, and clear goals are an important part of successful teamwork (Demiris et al., 2008; Jünger et al., 2007; Seow & Bainbridge, 2018).

*Professional contribution* in interprofessional teamwork is seen as educational concurrency, a clear role distribution and a common understanding of responsibilities. In educational concurrency, different professionals with different educational background participate in patient care (Leboul et al., 2017) in order to provide a more holistic form of care from several perspectives (Mélin et al., 2020). Overall, a diversity of professionals in a team creates the possibility of providing better care (Goebel et al., 2016) however, each profession might also wish to affect the prioritization of the care (Washington et al., 2017a, 2017b). Professionals might have different contributions (Wittenberg-Lyles et al., 2010) and expectations of the focus and objectives (Demiris et al., 2008). A clear role distribution (Brennan et al., 2016; Demiris et al., 2008; Mélin et al., 2020; Seow & Bainbridge, 2018), and clearly defined professional boundaries (Klarare et al., 2013; O'Connor & Fisher, 2011) in an interprofessional team ensures more confidence (Jünger et al., 2007) and will better engage professionals in the teamwork (Wittenberg-Lyles et al., 2010). A common understanding of responsibilities is that each profession in the team acknowledges their responsibilities (Klarare et al., 2013; Mélin et al., 2020), as well as their mutual responsibility for the patient’s care (Washington et al., 2017a). When responsibilities are shared evenly (Brennan et al., 2016) and tasks are well co-ordinated (Demiris et al., 2008) it has a positive effect on teamworking.

*Work management* in interprofessional teamwork in palliative care settings can be described as difficult as the work is time-consuming (Klarare et al., 2013; Washington et al., 2016, 2017a) and the workload is heavy (Klarare et al., 2013; Washington et al., 2017a). Too little time to collaborate will affect a professionals’ participation in interprofessional work (Klarare et al., 2013) and therefore time keeping is important (Brennan et al., 2016). Mostly teams have too many patient cases to deal with (Klarare et al., 2013; Washington et al., 2016) and often the length of meetings versus the number of patient cases are not in balance. This will affect both the patient’s care and the professionals’ working conditions (Washington et al., 2017a).

### Required Competence for Interprofessional Teamwork

**Figure 3.**
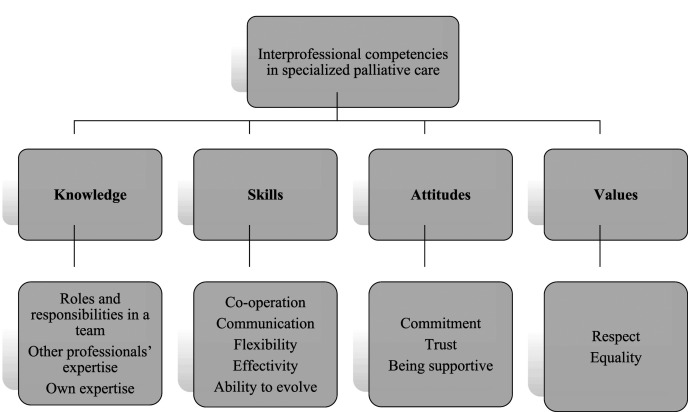
*Required competencies in interprofessional teamwork in specialized palliative care*.

Competence integrates knowledge, skills, attitudes, and values (Meretoja, 2003). Teamwork knowledge in palliative care consists of three subcategories: knowledge of *roles and responsibilities in a team, other professionals’ expertise, and own expertise* (Figure 3). Knowledge of *the professionals’ roles and responsibilities in a team* means identifying (Seow & Bainbridge, 2018), being aware of (Brennan et al., 2016; Mélin et al., 2020; Sundus et al., 2018) and understanding (Sundus et al., 2018) other professionals’ roles, and identifying their responsibilities (Mélin et al., 2020; Seow & Bainbridge, 2018). Knowledge of *other professionals´ expertise* means that members are aware of the teams’ resources (Brennan et al., 2016) as regards professional skills (Mélin et al., 2020; O'Connor & Fisher, 2011; Sundus et al., 2018), and expertise (Klarare et al., 2013; Sundus et al., 2018). Knowledge of one’s *own expertise* means that everyone should know their own roles and boundaries in a team (O'Connor & Fisher, 2011) and be confident in their own area of patient care (Klarare et al., 2013). It is important for individuals to complement the team with their own special expertise (Sundus et al., 2018), perform tasks well (Demiris et al., 2008) and understand their limits as a team member (Sundus et al., 2018).

Teamwork skills in palliative care consist of five subcategories: *co-operation, communication, flexibility, effectivity,* and *ability to evolve. Co-operation* involves both the ability to collaborate with other professionals (Goebel et al., 2016; Sundus et al., 2018; Wittenberg-Lyles et al., 2010), and also providing other professionals with the possibility to participate (Brennan et al., 2016) in the teamwork. Implementing the rules of the team is part of the co-operation in a team (Brennan et al., 2016). It is important to understand that there is no teamwork without *communication* (Jünger et al., 2007; Klarare et al., 2013). Communication in interprofessional teams should be organized (Washington et al., 2017b) and efficient (Washington et al., 2017a), because the time allowed for collaboration is usually limited and the number of participating professionals and manageable caseloads are high. Facilitating open (Jünger et al., 2007; Seow & Bainbridge, 2018) and frank (Demiris et al., 2008) communication in a team is important. Decisions should be made together in a team (Leboul et al., 2017; Sundus et al., 2018) and professionals should utilize other team members’ expertise when making care decisions (Leboul et al., 2017). Communication should be both formal and informal (Seow & Bainbridge, 2018) and often needs to be improved (Demiris et al., 2008). *Flexibility* (Brennan et al., 2016; Jünger et al., 2007) and patience (Seow & Bainbridge, 2018) are generally important in interprofessional teamwork. For example, the ability to make compromises as a part of a team when differing views are expressed is a one way to be flexible (Leboul et al., 2017). When working in interprofessional teams, members should accept the possibility of several opinions on a current topic (Jünger et al., 2007; Leboul et al., 2017) and to be open to changes and new ideas (Brennan et al., 2016; Mélin et al., 2020). Decisions that have been made should be amenable to the whole team (Jünger et al., 2007). To be *effective* (Brennan et al., 2016; Demiris et al., 2008; Washington et al., 2017a) and efficient means to be organized (Brennan et al., 2016) and focus on relevant information for example in interprofessional team meetings (Washington et al., 2017a). Effectivity is also the ability to maintain schedules when time is restricted (Brennan et al., 2016). The *Ability to evolve* as a team and as an individual means a group must develop into a team (Demiris et al., 2008); this can be accomplished by learning from mistakes, being willing to change working practices (Seow & Bainbridge, 2018) and by developing new ways of working together (Jünger et al., 2007).

Teamwork attitudes in palliative care consist of three subcategories: *commitment, trust* and *being supportive*. *Commitment* in teamworking means taking an interest in the work of the team (Seow & Bainbridge, 2018), having a positive attitude (Jünger et al., 2007), showing active participation (Washington et al., 2017a), and making an effort while working in an interprofessional team (O'Connor & Fisher, 2011). Commitment means (Demiris et al., 2008; Washington et al., 2017a) being committed to the common goal (Seow & Bainbridge, 2018) and accepting and implementing the teams’ rules (Brennan et al., 2016). Professionals should *trust* (Demiris et al., 2008) and rely on the team and other professionals (Jünger et al., 2007; O'Connor & Fisher, 2011) to work collaboratively. This often means trusting in other professionals’ expertise (O'Connor & Fisher, 2011; Sundus et al., 2018) in their own field of care. *Being supportive* means providing support (Demiris et al., 2008; Jünger et al., 2007; Klarare et al., 2013; Leboul et al., 2017; Seow & Bainbridge, 2018) and being supported by the team (Demiris et al., 2008; Jünger et al., 2007) in a team, it is every members’ responsibility to be as collaborative as possible.

Teamwork values in palliative care consist of two subcategories: *respect* and *equality*. To *respect* other professionals (Jünger et al., 2007; Sundus et al., 2018; Washington et al., 2017a) means understanding that every team member is important, and their opinion is needed when making decisions about a patient’s care (Washington et al., 2016). All professionals should feel valued (Washington et al., 2017a) and no disrespectful behavior should be allowed in a team (Jünger et al., 2007). Moreover, humility is needed in order to recognize other professionals’ contributions (Mélin et al., 2020). Implementing *equality* in a team means understanding that every professional has equal status in an interprofessional team (Washington et al., 2016, 2017a) and all the team members are important.

## Discussion

The aim of this literature review was to describe interprofessional teamwork in specialized palliative care settings and to describe the required competencies for teamwork. Overall, the description in this study of interprofessional teamwork and required competencies had similar features to other palliative care (Connolly et al., 2016; Gamondi et al., 2013) and interprofessional frameworks (CIHC, 2010; D’Amour et al., 2005; IPEC, 2016; WHO, 2010; Wilhelmsson et al., 2012; Wood et al., 2009). The results of this review were also in line with the WHOs guidelines (2016) about the importance of interprofessional teamworking in order to provide high-quality palliative care. However, this review reveals a few special aspects we would like to emphasize.

When patients and their families are facing life-threatening health conditions ethical aspects of care are strongly present, probably in a larger sense than other fields of care. According to the results with interprofessional teamwork it is possible to provide ethical, individual, and holistic care to patients with complex care needs in specialized palliative care (Klarare et al., 2013; Leboul et al., 2017; Mélin et al., 2020; Sundus et al., 2018). When planning the participation of different professionals in patient care, it is important that right until the end the care should be tailored to the patients’ individual needs (Leboul et al., 2017; Washington et al., 2017a, 2017b) and patients are viewed as individuals (Brennan et al., 2016). The importance of different professionals communicating, counseling (Sundus et al., 2018; Washington et al., 2017b), and devoting time, or simply being present (Mélin et al., 2020) cannot be overemphasized in palliative care when a prognosis cannot be given. In palliative care the presence of death also has an effect on professionals working in an interprofessional team. Every professional has their own individual way of confronting death and coping with the feeling’s death causes (Jünger et al., 2007; Klarare et al., 2013; Leboul et al., 2017), which should be acknowledged when working as a team in palliative care. It is known that palliative care has been changing toward more collaborative practices and targeting a holistic approach (Hui et al., 2018) rather than highlighting the medical-centered atmosphere in a team (Buss et al., 2017). In sensitive area like palliative care, it is important that the interprofessional team share the same philosophy (Jünger et al., 2007), and have a common vision about the goal of teamwork (Brennan et al., 2016; Jünger et al., 2007; Seow & Bainbridge, 2018). Cohesion in an interprofessional team will assist both the patient as well as the professionals working in a team. It is understandable that all professionals bring their own specialties to patient care (Wilhelmsson et al., 2012); however, the team dynamics are at least as important in palliative care as the overall health and social care.

Being a competent co-worker in an interprofessional team is the key to better collaborative practices and also in interprofessionally provided palliative care. In this review, interprofessional competence was described, according to Meretoja (2003), as knowledge, skills, attitudes, and values. The interprofessional competencies described in this review are common to all professionals participating in teamwork. These competencies might also be partially similar when compared to the discipline specific competencies in palliative care (Kang et al., 2013). Every professional has their own important role as a team member and interprofessionally implemented patient care. They should recognize their own role in relation to other professionals (Brennan et al., 2016; Mélin et al., 2020; Seow & Bainbridge, 2018; Sundus et al., 2018) and vice versa to provide individual and effective care. Professionals equipped with the skills of flexibility (Brennan et al., 2016; Jünger et al., 2007; Leboul et al., 2017), communication (Demiris et al., 2008; Jünger et al., 2007; Klarare et al., 2013; Seow & Bainbridge, 2018; Sundus et al., 2018; Washington et al., 2017a, 2017b) and co-operation (Brennan et al., 2016; Goebel et al., 2016; Sundus et al., 2018; Wittenberg-Lyles et al., 2010) will lead the team to the successful teamwork described earlier. The correct attitudes in teamworking, such as relying on other professionals’ expertise (Jünger et al., 2007; O'Connor & Fisher, 2011; Sundus et al., 2018) and providing support to others (Demiris et al., 2008; Jünger et al., 2007; Klarare et al., 2013; Leboul et al., 2017; Seow & Bainbridge, 2018) will benefit both, patients, and professionals. Implementing equality (Washington et al., 2016, 2017b) and respecting other professionals (Jünger et al., 2007; Mélin et al., 2020; Sundus et al., 2018; Washington et al., 2016, 2017b) in a team, could be the way to reduce the medical-centered atmosphere in palliative care (Buss et al., 2017). In the results of this review, knowledge and skills were highlighted. This might be because none of the included studies were directly describing required interprofessional competencies in palliative care. However, the teamwork description strongly included the ethical aspects.

Despite the active roles of professionals, it was previously known (Wilhelmsson et al., 2012) and verified by this review that actions are needed to sustain collaborative working in teams. Healthcare organizations and their management have a great responsibility to create and sustain a collaborative atmosphere. More attention should be paid to how teamwork is organized, and which professionals should participate in teamworking to provide individual care. This review proposes that the organizational culture, where each profession has their own meetings and support groups, should be discontinued and a collaboration implemented that is in- and outside of formal working situations. It is acknowledged that professionals need continuous training to be competent co-workers (IPEC, 2016); in addition, professionals should be given opportunities also in continuing education to learn in actual teams. The challenge for the future will be maintaining collaborative practices in demanding specialized palliative care settings in order to provide holistic care by competent professionals for an increasing number of palliative care patients.

### Recommendations for Future Research

• Future research should be more directed to the implementation of collaborative practices in palliative care facilities from the point of view of patients and family members.
• The perceptions of patients and family members should be studied in order to acquire a deeper understanding of the teamwork requirements and to provide more interprofessionally conducted holistic and individual palliative care.
• More knowledge is needed about interventions so as to increase interprofessional competence and measure levels of competence. In addition, more quantitative research is necessary, since mainly qualitative approaches have been utilized.

### Clinical Implications for Health Managers and Policymakers

• In order to provide better care outcomes, more attention should be paid to the working methods and the quality of collaborative practices in palliative care instead of concerns about the number of professionals.
• When confronting death and dying the way in which professionals deal with their own feelings has an effect on the whole team’s performance. Therefore, systematized supervision practices are essential in palliative care to support professionals in coping with their work.
• To increase the competence of professionals, it is important to develop a collaborative atmosphere and the value of teamwork in both professional and continuing education.

### Limitations

There are some limitations to this review. First, although interprofessional teamwork have been widely studied in healthcare context, there is lot of variation in terms used to describe the same phenomenon and they are not all clearly defined. This led to difficulties in forming the search phrases and defining appropriate terms for the literature search. Yet, the topic of this study is little studied; studies describing teamwork in specialized palliative care were included in this review even if the used concepts were not defined. Because of these reasons the expertise of two information specialists was used to process the search. Second, in this study the scope was on specialized palliative care, where the professionals are more educated and qualified as regards facing patients with life-limiting health conditions. It is possible that all of the selected studies were not conducted at target level of care because there might be variation in care delivery, and also in used terms between countries. By conducting the study evaluation by two reviewers this aspect was dealt (if needed, a third opinion was requested). Third, the studies were mainly conducted in North America and describe palliative care and teamworking in a certain geographical area. This might have affected the results of this review. Finally, the analysis process in content analysis is always subjective. Therefore, the results are dependent on the interpretation of the researcher and completely objective results are impossible to achieve.

## Conclusion

This review provided a description of how collaboration among health and social care professionals with different educational backgrounds can be achieved in specialized palliative care. Patients and their families profit from interprofessional teamwork by receiving ethical, individual, and holistic care to palliative. The nature of the provided care and the fact that the care relationship inevitably ends in the patients’ death also has an impact on interprofessional teamwork. More attention should be paid to supervision practices, but also working methods and the quality of collaborative practices, when taking care of palliative care patients with a life-limiting health condition. Ascertaining the competence of team members will produce better interprofessional practices in palliative care settings and increase the care outcomes. The findings of this review can be used as a framework when developing interventions to promote clinical and educational practices regarding interprofessional teamwork in palliative care.
